# Privacy-Preserving Semantic Segmentation Using Vision Transformer

**DOI:** 10.3390/jimaging8090233

**Published:** 2022-08-30

**Authors:** Hitoshi Kiya, Teru Nagamori, Shoko Imaizumi, Sayaka Shiota

**Affiliations:** 1 Department of Computer Science, Tokyo Metropolitan University, 6-6 Asahigaoka, Hino-shi, Tokyo 191-0065, Japan; 2 Graduate School of Engineering, Chiba University, 1-33 Yayoicho, Chiba 263-8522, Japan

**Keywords:** semantic segmentation, vision transformer, segmentation transformer, privacy-preserving

## Abstract

In this paper, we propose a privacy-preserving semantic segmentation method that uses encrypted images and models with the vision transformer (ViT), called the segmentation transformer (SETR). The combined use of encrypted images and SETR allows us not only to apply images without sensitive visual information to SETR as query images but to also maintain the same accuracy as that of using plain images. Previously, privacy-preserving methods with encrypted images for deep neural networks have focused on image classification tasks. In addition, the conventional methods result in a lower accuracy than models trained with plain images due to the influence of image encryption. To overcome these issues, a novel method for privacy-preserving semantic segmentation is proposed by using an embedding that the ViT structure has for the first time. In experiments, the proposed privacy-preserving semantic segmentation was demonstrated to have the same accuracy as that of using plain images under the use of encrypted images.

## 1. Introduction

Deep learning has been deployed in many applications including security-critical ones such as biometric authentication and medical image analysis. Generally, data contains sensitive information, so privacy-preserving methods for deep learning have become an urgent problem. In particular, data including sensitive information cannot be transferred to untrusted third-party cloud environments even if they provide a powerful computing environment. Therefore, it has been challenging to test a deep learning model in cloud environments while preserving privacy. To address the privacy issue, researchers have proposed various solutions. However, cryptographic methods such as fully homomorphic encryption are still computationally expensive [1,2,3], and moreover, the encrypted images cannot be directly applied to models trained with plain images. To protect privacy, federal learning (FL) has also been studied as a type of distributed machine learning [4,5]. FL is capable of significantly preserving clients’ private data from being exposed to adversaries. However, FL aims to construct models over multiple participants without directly sharing their raw data, so the privacy of input (query) images is not considered. For these reasons, numerous learnable perceptual encryption methods have been studied so far for various applications [6,7,8,9,10,11] that have been inspired by encryption methods for privacy-preserving photo cloud sharing services [12]. Most perceptual image encryption methods aim to realize the secure transmission/storage of images as in [13]. In contrast, learnable image encryption is encryption that allows us not only to generate visually protected images to protect personally identifiable information included in an image such as an individual or the time and location of the taken photograph but to also apply encrypted images to a machine learning algorithm in the encrypted domain.

Perceptual encryption-based methods have been verified to be effective in image classification tasks [7,8,9,14], but other tasks such as semantic segmentation have never been considered under the use of perceptual encryption-based methods because such tasks are required to achieve a pixel-level accuracy [15]. Accordingly, in this paper, we propose a novel method for privacy-preserving semantic segmentation that uses encrypted images and models with the vision transformer (ViT) [16], called the segmentation transformer (SETR) [17]. The reason that we focus on ViT–based models is given below. ViT is well known to have a higher performance than conventional convolutional neural networks (CNNs) in some settings. In addition, following the success of ViT in image classification tasks, it is expected to have success in other tasks including semantic segmentation, and ViT also has an embedding structure. In particular, the embedding structure plays an important role in the proposed method. In this paper, we point out for the first time that embedding enables us not only to avoid the influence of block-wise encryption but to also update a secret key easily in a semantic segmentation task, which conventional methods cannot.

We make the following contributions in this paper.

- We propose the combined use of encrypted images and models in a semantic segmentation task to protect visual sensitive information of input images for the first time.
- We confirm that the proposed method allows us not only to use the same accuracy as that when images are not encrypted but to also update a secret key easily.

In addition, the proposed method does not need any network modification. If other models with an embedding structure are developed, the proposed method is also expected to be effective under the use of these models as well.

The rest of this paper consists of related work, the proposed privacy-preserving semantic segmentation, experiments, and a discussion before the conclusion.

## 2. Related Work

### 2.1. Privacy-Preserving DNNs

Privacy-preserving machine learning methods with homomorphic encryption (HE) [1,18,19,20,21,22] have been studied. One is CryptoNet [21], which can apply HE to the influence stage of DNNs. CryptoNet has very high computational complexity, so a dedicated low computer convolution core architecture for CryptoNet was proposed and implemented with CMOS technology [22]. In CryptoNet, all activation functions and the loss function must be polynomial functions. Therefore, privacy-preserving machine learning methods with HE are still difficult to be applied to state-of-the-art DNNs.

In comparison, an approach with HE was proposed for privacy-preserving weight transmission for multiple owners who wish to apply a machine learning method over combined data sets [1,18,19,20]. In this approach, since the gradients are encrypted by using HE, model information is not leaked. The privacy-preserving weight transmission can provide robustness against model extraction attacks. However, this approach does not aim to protect sensitive information of input (query) images. To protect privacy, federal learning (FL) has also been studied as a type of distributed machine learning [4,5]. FL is capable of significantly preserving clients’ private data from being exposed to adversaries. Clients store training data locally and use the data to train a local model. Then, the clients upload the trained parameters to a server. In this way, each client can collaboratively train one model on the server protecting the privacy of the data. However, FL aims to construct models over multiple participants without directly sharing their raw data, so the privacy of input images is not considered. The proposed method allows us to protect sensitive information of input images without any performance degradation.

### 2.2. Learnable Image Encryption for Machine Learning

Learnable encryption encrypts images with a secret key so that visual information in encrypted images is not perceptible to humans while maintaining the ability to classify encrypted images with a model, where a model is trained by using encrypted images. The first concept was introduced for traditional machine learning such as support vector machine (SVM) and random forests [23,24,25]. For deep learning, Tanaka first introduced block-wise learnable image encryption (LE) with an adaptation layer that is used prior to the classifier to reduce the influence of image encryption [7]. Another encryption method is pixel-wise encryption (PE) in which negative-positive transformation and color component shuffling are applied without using any adaptation layer [9]. However, both block-wise and pixel-wise encryption methods can be attacked by ciphertext-only attacks [6,26]. To enhance the security of encryption, Tanaka’s method was extended by adding a block-scrambling step and utilizing different block keys for the pixel encryption operation [8] (hereinafter denoted as ELE). In addition, to improve both the security of encryption and the accuracy of models, the use of isotropic networks such as ViT has been investigated [27,28]. However, there are still several issues: lower accuracy than models trained with plain images, unknown applicability to semantic segmentation, and the lack of an update method for the key. Accordingly, we aim to overcome these issues in this paper.

### 2.3. Segmentation Transformer

**Figure 1:**
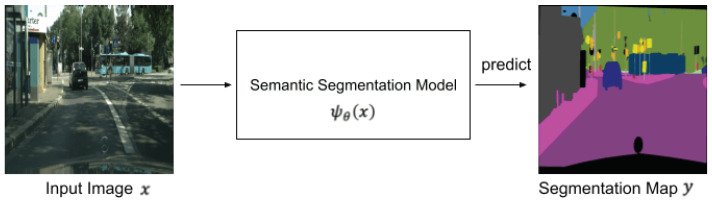
Overview of semantic segmentation.

The purpose of semantic segmentation is to classify objects in a pixel-level resolution. Figure 1 shows an overview of semantic segmentation. A segmentation model predicts a segmentation map from an input image, where each pixel in the segmentation map represents a class label. The segmentation transformer (SETR) is the first transformer-based model proposed for semantic segmentation [17]. This model is inspired by ViT, which has a high performance in image classification tasks.

**Figure 2:**
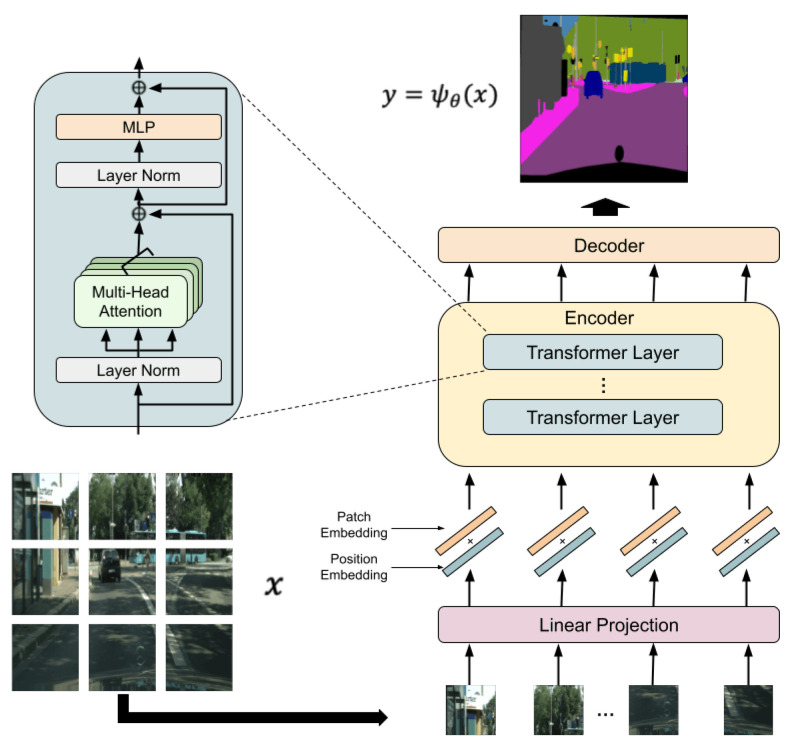
Architecture of segmentation transformer [17].

Figure 2 shows the architecture of SETR, where the encoder is the same as in ViT. Since the encoder in ViT receives only 1-D vectors as an input, an image is divided into patches. Then, each patch is flattened and converted to a 1-D vector. Two embeddings are used to understand the location information of separated patches in an original image, and each patch is mapped to learnable dimensions. The former, called position embedding, embeds location information about where each patch is in an original image. The latter, called patch embedding, maps each patch to learnable dimensions using a matrix. By using these two types of embeddings, learning is possible even when an image is divided into patches while keeping location information [27]. After that, the feature representation obtained by the encoder is inputted to the decoder, which outputs a segmentation map of the same size as an input image. The proposed method is carried out on the basis of the embedding structure.

## 3. Proposed Method

### 3.1. Overview and Threat Model

**Figure 3:**
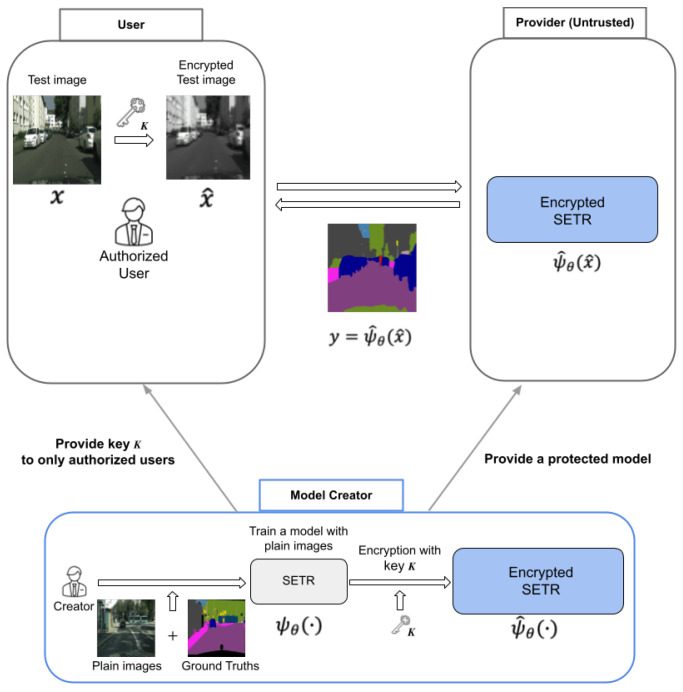
Overview of privacy-preserving semantic segmentation. (SETR: segmentation transformer).

Figure 3 shows an overview of the proposed method for protecting visual information on plain images for semantic segmentation. The method is summarized as below.

First, a model creator trains a model with plain images. Next, after training, the model $\psi_{\theta }\left(\cdot \right)$ is encrypted with a secret key *K* as ${\hat{\psi }}_{\theta }\left(\cdot \right)$. Then, key *K* is provided to authorized users, and the protected model is provided to a provider, who has no key. Then, the users encrypt query (input) images with key *K* to protect sensitive information included in the images and send the encrypted ones to the server. Finally, the users receive segmentation maps from the server. The main purpose of this work is to protect sensitive information of the input images the users have. The proposed method can be carried out without both any performance degradation and network modification, compared with the use of plain images.

As we focus on protecting personally identifiable information included in an image such as an individual or the time and each car’s number in a semantic segmentation scenario, the goal of an adversary is to illegally restore such personally identifiable information. Encrypted images are transferred to an untrusted provider for testing query data as in Figure 3. In this paper, we assume the adversary knows the encryption algorithm but not the secret key. In other words, we assume that the adversary can carry out a ciphertext-only attack (COA) by using only query images that the adversary receives, as in conventional perceptual encryption methods [6].

### 3.2. Encryption Method

ViT utilizes patch embedding and position embedding. In this paper, we use a unique property of the embedding structure. In SETR, an image tensor $x\in R^{h\times w\times c}$ is divided into *N* patches with a size of p×p where *h*, *w* and *c* denote the height, width, and the number of channels of an image. When *h* and *w* are dividable by *p*, *N* is given as $hw/p^{2}$. After that, each patch is flattened as $x_{p}^{i}=\left[x_{p}^{i}\left(1\right),x_{p}^{i}\left(2\right),\ldots ,x_{p}^{i}\left(L\right)\right]$, and the resulting 1-D vector is linearly mapped to a vector with dimensions of *D* with a learnable matrix **E** as

(1) $$\begin{array}{cc} z_{0}= & \left[x_{class};x_{p}^{1}E;x_{p}^{2}E;\cdots x_{p}^{i}E;\cdots x_{p}^{N}E\right]+E_{\mathrm{pos}}, \end{array}$$

where

$$\begin{array}{cc} E_{\mathrm{pos}}= & {\left({\left(e_{pos}^{0}\right)}^{⊤}{\left(e_{pos}^{1}\right)}^{⊤}\cdots {\left(e_{pos}^{i}\right)}^{⊤}\cdots {\left(e_{pos}^{N}\right)}^{⊤}\right)}^{⊤},\\ E\in & R^{L\times D},\;E_{\mathrm{pos}}\in R^{(N+1)\times D},\\ x_{p}^{i}\in & R^{L},\;e_{pos}^{i}\in R^{D},\;L=p^{2}c, \end{array}$$

$x_{class}$ is the classification token which is the input to MLP (see Figure 2), $e_{pos}^{0}$ is the information of the classification token, $e_{pos}^{i}$ is the position information of each patch, and $z_{0}$ is the embedded patch. **E** and $E_{\mathrm{pos}}$ are decided by training a model with plain images. By using a trained model $\psi_{\theta }$, a segmentation map *y* is given by

(2) $$y=\psi_{\theta }\left(x\right).$$

The proposed method is carried out in accordance with the above relation.

#### 3.2.1. Model Encryption

In the proposed method, trained models have to be encrypted to use encrypted input images. A matrix **E** is transformed with key *K* after training a model as follows.

(1) Randomly generate a matrix $E_{\mathrm{enc}}$ with key *K* as
  (3) $$\begin{array}{c} E_{\mathrm{enc}}=\left[\begin{array}{cccc} k_{\left(1,1\right)} & k_{\left(1,2\right)} & \cdots & k_{\left(1,L\right)}\\ k_{\left(2,1\right)} & k_{\left(2,2\right)} & \cdots & k_{\left(2,L\right)}\\ \vdots & \vdots & \ddots & \vdots \\ k_{\left(L,1\right)} & k_{\left(L,2\right)} & \cdots & k_{\left(L,L\right)} \end{array}\right], \end{array}$$
  $$\begin{array}{cc} k_{\left(i,j\right)} & \in R,\;i,j\in \left\{1,\cdots ,L\right\},\\ E_{\mathrm{enc}} & \in R^{L\times L},\mathrm{and}\;\det E_{\mathrm{enc}}\neq 0. \end{array}$$
(2) Multiply $E_{\mathrm{enc}}$ and **E** to obtain $\hat{E}$ as
  (4) $$\hat{E}=E_{\mathrm{enc}}E,\;\hat{E}\in R^{L\times D}.$$
(3) Replace **E** in Equation (1) with $\hat{E}$ as a new patch embedding to encrypt a model.

Namely, an encrypted model ${\hat{\psi }}_{\theta }$ is given by

(5) $${\hat{\psi }}_{\theta }=t\left(\psi_{\theta },E_{\mathrm{enc}}\right),$$

where t(·) is the proposed model encryption algorithm.

#### 3.2.2. Example of $E_{\mathrm{enc}}$

There is a lot of flexibility in the design of $E_{\mathrm{enc}}$. A design example of $E_{\mathrm{enc}}$ is given as follows.

(1) Generate a random integer vector with a length of *L* by using a random generator with a seed value as
  (6) $$l_{enc}=\left[l_{e}\left(1\right),l_{e}\left(2\right),\ldots ,l_{e}\left(i\right)\;,\ldots ,l_{e}\left(L\right)\right]\;,$$
  where
  $$\begin{array}{cc} le(i)\in & \left\{1,2,...,L\right\},\\ le(i)\neq & le(j)\;\mathrm{if}\;i\neq j\;. \end{array}$$
(2) Decide $k_{\left(i,j\right)}$ in Equation (3) with $l_{enc}$ as

(7) $$k_{\left(i,j\right)}=\left\{\begin{array}{cc} 0 & \left(j\neq l_{e}\left(i\right)\right)\\ 1 & \left(j=l_{e}\left(i\right)\right) \end{array}\right.\;.$$

For example, if *L* = 4 and $l_{enc}$ = (4,1,3,2), $E_{\mathrm{enc}}$ is given by

(8) $$\begin{array}{c} E_{\mathrm{enc}}=\left[\begin{array}{cccc} 0 & 0 & 0 & 1\\ 1 & 0 & 0 & 0\\ 0 & 0 & 1 & 0\\ 0 & 1 & 0 & 0 \end{array}\right]. \end{array}$$

In addition, $E_{\mathrm{enc}}$ in Equation (8) is an orthogonal matrix, so the relation,

(9) $$E_{\mathrm{enc}}^{-1}=E_{\mathrm{enc}}^{⊤},$$

is satisfied where ${}^{⊤}$ is a transposition. Key *K* corresponds to a seed value used in a random integer generator.

#### 3.2.3. Test Image Encryption

We assume that an authorized user has key *K*, so the user can generate $E_{\mathrm{enc}}$ by using *K* as well. Accordingly, a new matrix ${\hat{E}}_{\mathrm{enc}}$ can be produced by

(10) $${\hat{E}}_{\mathrm{enc}}=E_{\mathrm{enc}}^{-1}.$$

An encrypted test image $\hat{x}\in R^{h\times w\times c}$ is produced by an authorized user as follows.

(a) Divide a test (query) image tensor $x\in R^{h\times w\times c}$ into blocks with a size of p×p such that $B=\left\{B_{1},\cdots ,B_{N}\right\}$.
(b) Flatten each block $B_{i}$ into a vector $b_{i}$ such that
  (11) $$b_{i}=\left[b_{i}\left(1\right),\cdots ,b_{i}\left(L\right)\right],$$
(c) Generate an encrypted vector $\hat{b_{i}}$ by multiplying $b_{i}$ by ${\hat{E}}_{\mathrm{enc}}$ as
  (12) $$\hat{b_{i}}=b_{i}{\hat{E}}_{\mathrm{enc}},\;\hat{b_{i}}\in R^{L},$$
(d) Concatenate the encrypted vectors into an encrypted test image $\hat{x}$.

As a result, when $\hat{x}$ is applied to the encrypted model, the embedded patch becomes

(13) $$\begin{array}{cc} z_{0}= & \left[x_{class};{\hat{x}}_{p}^{1}\hat{E};{\hat{x}}_{p}^{2}\hat{E};\cdots {\hat{x}}_{p}^{i}\hat{E};\cdots {\hat{x}}_{p}^{N}\hat{E}\right]+E_{\mathrm{pos}},\\ = & \left[x_{class};x_{p}^{1}E;x_{p}^{2}E;\cdots x_{p}^{i}E;\cdots x_{p}^{N}E\right]+E_{\mathrm{pos}}, \end{array}$$

where ${\hat{x}}_{p}^{i}$ is the *i*-th patch of an encrypted test image.

Accordingly, when $\hat{x}$ is inputted to an encrypted model ${\hat{\psi }}_{\theta }$, an output $\hat{y}$ is given by

(14) $$\begin{array}{cc} \hat{y}= & {\hat{\psi }}_{\theta }\left(\hat{x}\right)\\ = & \psi_{\theta }\left(x\right)=y. \end{array}$$

From Equation (14), the combined use of encrypted image $\hat{x}$ and encrypted model ${\hat{\psi }}_{\theta }$ can give the same output as that of plain model $\psi_{\theta }$.

When Equations (7) and (8) are chosen as $E_{\mathrm{enc}}$, ${\hat{E}}_{\mathrm{enc}}$ is given by

(15) $$\begin{array}{cc} {\hat{E}}_{\mathrm{enc}}= & \left[\begin{array}{cccc} 0 & 1 & 0 & 0\\ 0 & 0 & 0 & 1\\ 0 & 0 & 1 & 0\\ 1 & 0 & 0 & 0 \end{array}\right]. \end{array}$$

This selection corresponds to pixel shuffling (SHF) in each block.

### 3.3. Requirements of Proposed Method

The proposed method satisfies the following requirements.

(a) Semantic segmentation can be carried out by using visually protected input images without sensitive information.
(b) No network modification is required.
(c) A high accuracy, which is close to that of using plain images, can be maintained.
(d) Keys are easily updated.

Requirement (a) is the main purpose of this work. As described in Section 3.2, the proposed method encrypts trained models on the basis of a matrix transformation, so there is no need to modify the structure of models. In addition, under Equation (10), the combined use of the encrypted images and model can produce the same result as that without any encryption. When we want to update key *K*, the model is easily updated by using a new $E_{\mathrm{enc}}$ generated with a new key. Accordingly, the proposed method can satisfy all the above requirements.

Although many privacy-preserving DNNs have been studied so for, some of them focus on privacy-preserving model training [1,4,5,18,19,20]. Numerous methods can protect the visual information of input images, but the methods are not capable of semantic segmentation tasks [6,7,8,9,10,11,12,14]. In addition, they degrade the performance of models compared with that without any encryption. Accordingly, the proposed method enables to satisfy all the above requirements for the first time.

## 4. Experimental Results

### 4.1. Setup

We conducted semantic segmentation experiments on two datasets to verify the effectiveness of the proposed method. The first dataset was Cityscapes [29], which is an urban scene dataset with 19 object categories. It consists of 5000 images with a resolution of 2048 × 1024 in total, and the images were divided into 2975, 500, and 1525 sets for training, validation, and testing, respectively. The other dataset was ADE20K [30], which is a benchmark for scene analysis with 150 categories. It consists of 20,210, 2000, and 3352 images for training, validation, and testing, respectively. In the experiments, training and validation images were used for training and testing, respectively. These two datasets used in this experiment are the same as in the paper [17] in which SETR was proposed. In this paper, the effectiveness of our method was confirmed by using these datasets. The effectiveness of the method does not depend on datasets.

In SETR [17], the encoder has two variations: T-base and T-large. T-base is a small model with 12 transformer layers and 768 hidden layers, and T-large is a large model with 24 transformer layers and 1024 hidden layers. In addition, there are three types of decoders: *Naïve*, which employs a simple two-layer network followed by a bilinear upsampler to restore an original resolution, *PUP*, which alternates between convolution layers and upsampling operations, and *MLA*, which uses multi-stage features such as a feature pyramid network.

In the experiment, all types of decoders were used on the two datasets. In addition, for Cityscapes, T-base was used as the encoder, and pre-trained weights of Deit [31] were utilized to initialize all transformer layers and input the linear projection layers of the model. For ADE20K, T-large was used as the encoder, and the pre-trained weights of ViT were utilized to initialize all transformer layers and input the linear projection layers of the model. In addition, input images were resized to images with a size of 768×768 for Cityscapes and to images with a size of 512×512 for ADE20K. Other training conditions including data augmentation methods used in the experiments were the same as those in [17].

As an evaluation metric, we used mean intersection-over-union (mIoU), which is an average of the intersection-over-union (IoU) for each class defined as

(16) $$IoU=\frac{TP}{TP+FP+FN},$$

where TP, FP, and FN mean true positive, false positive, and false negative values calculated from a predicted full-resolution output and ground truth, respectively. When a IoU value is closer to 1, it indicates a higher accuracy.

### 4.2. Semantic Segmentation Performance

**Figure 4:**
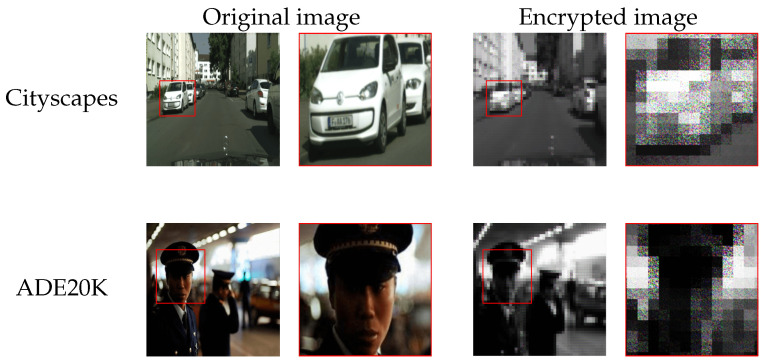
Example of encrypted images (p×p=16×16). Zoom-ins of red-framed regions are shown on right side of each image. The red boxes represent sensitive information such as license plates.

In the experiment, SETR models with a patch size of *p* = 16 were trained by using plain images, and then trained models were encrypted with a secret key *K* in accordance with the proposed procedure, where $E_{enc}$ was generated on the basis of Equations (6) and (7). In addition, test images were encrypted with key *K* as well. Figure 4 shows an example of encrypted images. From the figure, sensitive visual information in images was confirmed to be protected by using image encryption. Table 1 also shows experimental results for each decoder for the two datasets where baseline represents the results for the models without encryption, and Correct(*K*) represents the results when an authorized user applied images encrypted with key *K* to the encrypted models. From the table, the proposed method allows authorized users not only to protect sensitive information but to also gain the same performance from encrypted models as that from plain models.

**Figure 5:**
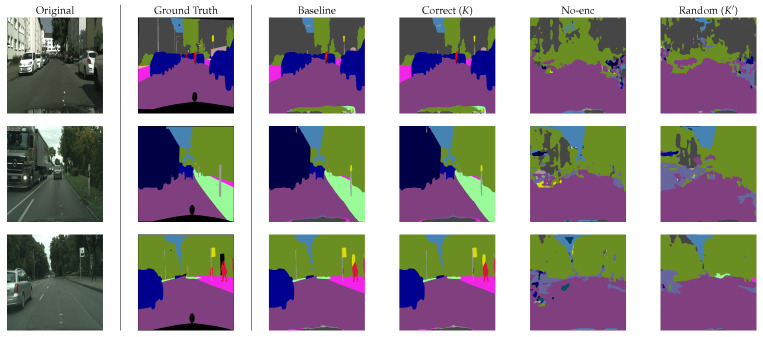
Example of predicted segmentation maps (with PUP on Cityscapes).

**Figure 6:**
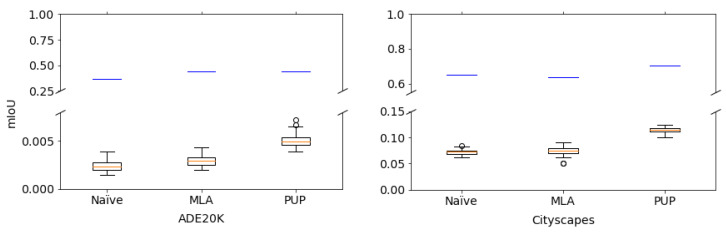
Mean IoU (mIoU) values of protected models with randomly generated 50 keys. Boxes span from first to third quartile, referred to as $Q_{1}$ and $Q_{3}$, and whiskers show maximum and minimum values in range of [$Q_{1}-1.5\left(Q_{3}-Q_{1}\right),Q_{3}+1.5\left(Q_{3}-Q_{1}\right)$]. Band inside box indicates median. Outliers are indicated as dots. Blue lines represent each baseline.

Random ($K^{\prime }$) and No-enc in Table 1 show the results for unauthorized users. In the experiment, we randomly generated 50 keys for Random ($K^{\prime }$), and the average value of 50 trials was computed under each condition. In addition, plain images were applied to encrypted images for No-enc. From the table, unauthorized users without key *K* could not gain high performance from the encrypted models. An example of predicted segmentation maps is given in Figure 5. From the figure, the effectiveness of the method was also verified. Figure 6 also shows the performance of the models in more detail when randomly generated 50 keys were used. The highest value of mIoU in the experiment was 0.13, which was still low.

### 4.3. Comparison with Conventional Methods

In this paper, we proposed a privacy-preserving semantic segmentation method to protect visually sensitive information of input images for the first time. Many conventional methods for privacy-preserving DNNs do not consider protecting sensitive information of input images such as federal learning [1,4,5,18,19,20]. Several methods can protect sensitive information of input images, in which encrypted images are used for model training, but the use of encrypted images for model training is known to degrade the performance of models [6,7,8,9,10,11,12,14]. In particular, for privacy-preserving semantic segmentation, the performance of models is heavily degraded in general because a pixel-level resolution is required for semantic segmentation [15]. In addition, when updating the key, the model has to be retrained by using images encrypted with a new key.

To compare the proposed method with conventional methods, Table 2 shows the results of CNN models trained with images encrypted by a block-wise encryption method, which was proposed in [32]. In [32], three encryption methods: pixel shuffling (SHF), negative/positive transformation (NP), and format-preserving Feistel-based encryption (FFX) were proposed. From the table, encrypting images to protect sensitive information in CNNs without embedding structures significantly degraded the accuracy of models compared to baseline due to the influence of encryption, even if the correct key was used. This problem is caused by the collapse of spatial information because CNNs do not have structures to store pixel location information. In contrast, the proposed method focuses on a transformer model that has embedding structures to preserve positional information, and thus the proposed method can maintain accuracy while protecting sensitive information in images.

### 4.4. Robustness against Attacks

Encrypted images have to be robust against attacks that aim to restore sensitive visual information from encrypted images. Numerous attack methods have been proposed to evaluate the robustness of perceptual encryption methods [26,33,34,35,36]. Existing learnable encryption methods have been evaluated under ciphertext-only attacks (COA). In this paper, to evaluate the robustness of encrypted images used in SETR, we considered brute-force attacks and the feature reconstruction attack (FR-Attack) [26], which exploits the local properties of an encrypted image to reconstruct visual information from encrypted images as a COA. Furthermore, as the distribution of the dataset is known, we also considered that the adversary may prepare exact pairs of plain images and encrypted ones with multiple different keys to learn a transformation model, i.e., the inverse transformation network attack (ITN-Attack) [34]. In addition, since images are encrypted by using a block-wise method, a jigsaw puzzle solver attack [35,36] was also used for evaluating the robustness of the proposed method.

(1) Key Space: The key space describes a set of all possible keys in an encryption algorithm. For the case where an image is divided into blocks with a size of p×p, the key space of the proposed algorithm (pixel shuffling) in Equation (7) is given as below.

(17) $$S_{key}=\left(p\times p\times c\right)!$$

For example, when p×p is chosen as the patch size of SETR, the key space is given by

(18) $$S_{key}=\left(16\times 16\times 3\right)!≃2^{6259}.$$

The use of a large key space enhances robustness against blue-force attacks. Typical cipher systems are recommended to have $2^{128}$ as a key space as in [37], so the proposed method has a large key space. Accordingly, it is expected to be robust against brute-force attacks.

**Figure 7:**
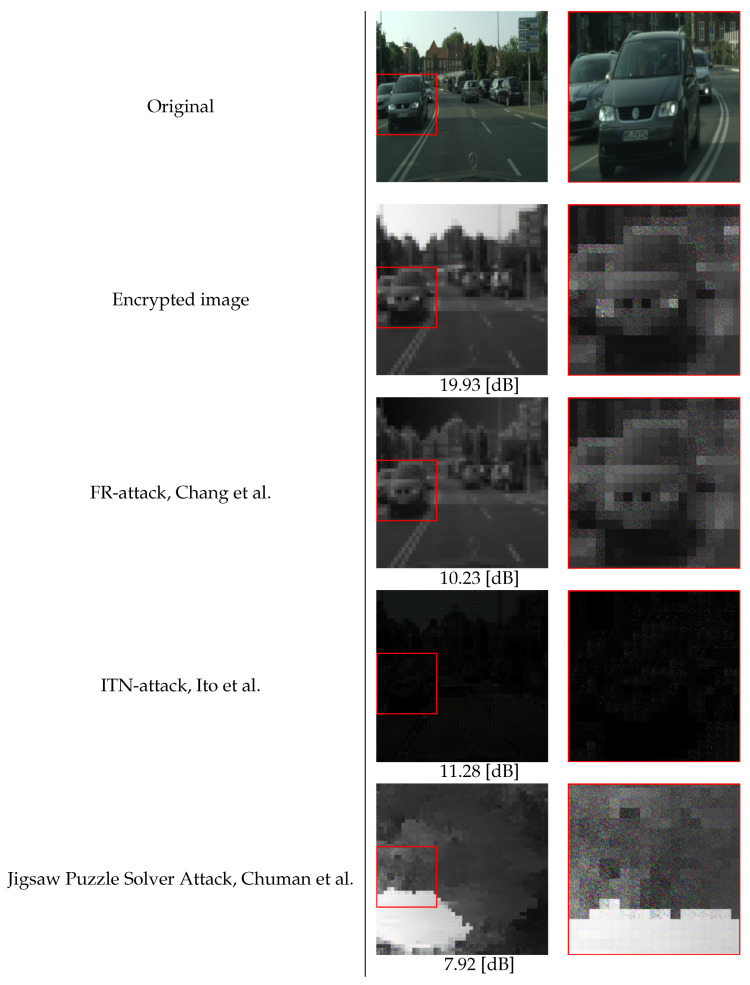
Examples of images restored from encrypted ones, see [26,34,35,36]. Zoom-ins of red-framed regions are shown on right side of each image. The red boxes represent sensitive information such as license plates. PSNR values are given under images.

(2) Robustness Against Attack Methods: Three state-of-the-art attack methods, FR-attack, ITN-attack, and jigsaw puzzle solver attack, were used for evaluation. Figure 7 shows images restored by using the three attacks. Peak signal-to-noise ratio (PSNR) values are marked at the bottom of the restored images to illustrate the perceived sensitivity of the noise component between a restored image and an original one. A larger value means less degradation between the two images. The results from Figure 7 indicate that the encrypted images with the proposed method did not have personally identifiable visual information in the original images even after the attacks. In addition, we also confirmed that the restored images for other test images in the test set followed a similar trend as in Figure 7. Therefore, the proposed method was robust against such attacks.

As another interesting attack, attackers may learn the transformation matrix between plain images and encrypted images to build a neural network to predict encrypted images given plain images, where attackers do not have the pairs of plain images and images encrypted with the correct key, so encrypted images have to be predicted by using randomly generated keys. Encrypted images can be predicted from plain images, but the images are different from images encrypted with the correct key. Thus, if these predicted images are applied to encrypted SETR, the accuracy of the estimated segmentation maps will be low as well as for random ($K^{\prime }$) in Table 1 and Figure 6. We carried out a preliminary experiment to confirm the validity of the above consideration. In addition, this attack cannot restore sensitive visual information from predicted images.

## 5. Conclusions

In this paper, we proposed the combined use of SETR, which is based on the vision transformer, and encrypted images for privacy-preserving semantic segmentation for the first time. The proposed method is carried out on the basis of the embedding structure that ViT has so that it enables us not only to protect sensitive visual information in plain images but to also use the same accuracy from encrypted models as that of models without encryption. Moreover, it does not need any network modification for privacy-preserving DNNs, and it is possible to easily update the key used for model encryption. In experiments, the proposed method was demonstrated to be effective in terms of segmentation accuracy and robustness against various attacks.

In this paper, the effectiveness of the proposed method was verified under the use of SETR, but the proposed method would be effective under the use of other models with an embedded structure. If models with an embedded structure that perform better than SETR are developed, the method is expected to get a higher performance under the use of these models while protecting sensitive information. As for future work, we shall investigate whether the proposed method can be extended to other networks with an embedding structure.

## Figures and Tables

**Table 1:** Accuracy of proposed models (mIoU).

Dataset	Selected Decoder	Baseline	Correct (*K*)	No-Enc	Random ($K^{\prime }$)
Cityscapes	*Naïve*	0.6490	0.6490	0.0674	0.0718
	*MLA*	0.6386	0.6386	0.0792	0.0743
	*PUP*	0.7039	0.7039	0.1135	0.1137
ADE20K	*Naïve*	0.3710	0.3710	0.0023	0.0024
	*MLA*	0.4370	0.4370	0.0030	0.0029
	*PUP*	0.4383	0.4383	0.0048	0.0050

**Table 2:** Accuracy (mIoU) of conventional method with encrypted images [15].

Network	Fully Convolutional Network (FCN)								
Block size	SHF			NP			FFX		
	Correct (*K*)	No-enc	Random ($K^{\prime }$)	Correct (*K*)	No-enc	Random ($K^{\prime }$)	Correct (*K*)	No-enc	Random ($K^{\prime }$)
4	0.4731	0.4536	0.3671	0.4706	0.3359	0.1505	0.3823	0.0157	0.0012
16	0.2214	0.1994	0.1150	0.3439	0.2114	0.0832	0.2611	0.0007	0.0079
Baseline	0.5966								

## Data Availability

The data presented in this study are available on request from the corresponding author.
